# Balancing fidelity and flexibility: a case study presentation of an augmented dynamic adaptation process for socio-technical innovations in healthcare

**DOI:** 10.3389/frhs.2026.1737047

**Published:** 2026-02-27

**Authors:** Suzanne S. Sullivan, Sharon Hewner, Sabrina Casucci, Elizabeth Bowen, Varun Chandola, Amy M. Sheehan, Amanda J. Anderson, Jarod Gabello, Katia Noyes

**Affiliations:** 1 College of Nursing, Upstate Medical University, State University of New York, Syracuse, NY, United States; 2 School of Nursing, University at Buffalo, State University of New York, Buffalo, NY, United States; 3 Department of Industrial Systems Engineering, University at Buffalo, State University of New York, Buffalo, NY, United States; 4 School of Social Work, University at Buffalo, State University of New York, Buffalo, NY, United States; 5 Department of Computer Science and Engineering, University at Buffalo, State University of New York, Buffalo, NY, United States; 6 National Center on Homelessness Among Veterans, United States Department of Veterans Affairs, Bronx, NY, United States; 7 Division of Health Services Policy and Practice, Department of Epidemiology and Environmental Health, School of Public Health and Health Professions, University at Buffalo, Buffalo, NY, United States

**Keywords:** cross-institutional teamwork, cross-sector care, cross-sector collaboration, failure to rescue, health information exchange, high needs, implementation science, quality improvement

## Abstract

**Introduction:**

The key *challenge* of successful implementation of healthcare innovations is balancing between intervention fidelity and the constantly changing healthcare environment where the implementation occurs. Unexpected changes as well as staff turnover are likely to affect implementation success and sustainability. Ensuring implementation fidelity requires researchers to adhere to the study design and implementation plan.

**Methods:**

We augmented the Dynamic Adaptation Process model using strategies from quality improvement, clinical safety, and software development agility to allow for continuous adaptation of the implementation process to changes in community healthcare settings and to reduce the chance of implementation failure when implementing complex socio-technical solutions in fast-paced healthcare environments.

**Results/discussion:**

This project illustrates how this augmented model could avert “implementation failure” based on real-life implementation cases of a socio-technical innovation to optimize cross-sector collaborations to support patients with medical, social and behavioral complexities. Key takeaways from the project highlight the importance of understanding baseline organizational readiness, designing for sustainability and spread, prioritizing engagement and communication, and ensuring a holistic design of socio-technical solutions.

## Introduction

Despite enthusiasm for translating evidence into practice, over 60% of implementation efforts fail (1–3). Complex interventions involving large teams, multiple agencies, or socio-technical innovations are at the greatest risk for implementation drift or failure, and most never become fully implemented or integrated into practice. Implementation failure results from interventions that are misaligned with preferences of key stakeholders or because implementation requires a substantial disruption of current practices.

Many implementation frameworks have the potential to optimize collaboration across clinical and data teams, reduce waiting times and healthcare costs, and improve outcomes. However, implementing socio-technical interventions is especially challenging, as a good fit requires establishing a careful balance between technological capabilities, user workflows, workforce skills, and a supportive organizational climate. This is especially challenging within the fast-paced, rapidly evolving healthcare environment.

We present two real-life case studies from the Personalized Cross-sector Transitional Care Management (PC-TCM) project to illustrate how an extension of the Dynamic Adaptation Process model (DAP) (4) into pre-implementation phases (conceptualization and design) can improve implementation success. By adding a formal reflective process, a Rapid Response Team, and clinical quality improvement strategies adapted from the patient safety concept, “Failure to Rescue,” we demonstrate how the modified DAP can avert “implementation failure” of socio-technical innovations. This manuscript follows the EQUATOR guidelines for reporting organizational case studies.

## Theoretical frameworks guiding dap modification

### The dynamic adaptation process (DAP) model

The DAP supports planned adaptation of evidence-based interventions by distinguishing core components from adaptable features, guiding allowable changes, training, fidelity monitoring, and system adjustments in a planned, rather than *ad hoc* way. The DAP model involves identifying core elements and adaptable characteristics of an evidence-based practice, then provides specific training on allowable adaptations, processes for fidelity monitoring and support, and identification of solutions to system and organizational adaptations. Thus, the DAP model improves real-world implementation fidelity while supporting iterative change.

### Designing for dissemination and sustainability (D4DS)

Following the Designing for Dissemination and Sustainability (D4DS) framework (5), we augmented the DAP model by engaging core stakeholders and initiating dynamic adaptation processes earlier upstream during the intervention development process, starting with conceptualization and intervention design. By doing so, we effectively extended the DAP from an implementation only study design to a hybrid effectiveness-implementation design. The original DAP model included four sequential phases (6). In our project, we extended DAP by allowing feedback from the early phases of the intervention design to inform subsequent phases of implementation and scale up.

## Materials and methods

The PC-TCM project is a 5-year implementation study to improve cross-sector collaboration for persons with high medical, social, and/or behavioral health needs. We received IRB approval from the State University of New York prior to implementing the project. The PC-TCM project partnered academic researchers with a Federally Qualified Health Center (primary care), a behavioral health organization, a social service agency providing temporary shelter, transitional housing, food, and related services to individuals in the community, and HEALTHeLINK, the regional health information exchange (HIE), in Buffalo, New York. The HIE connects hospitals, providers, and labs across health systems through shared health information. The primary care practice provides services for more than 26,173 patients annually, of whom only 5% are ages 65 and over, and 70% are primarily insured by Medicaid. The behavioral health organization serves over 6,700 individuals annually. HIEs are a federal initiative that enable secure electronic sharing of patient data across organizations aiming to improve coordination, reduce costs of care, and enhance patient safety (7, 8). Governed by privacy and interoperability regulations, HIEs operate under opt-in/opt-out patient consent models. HIEs have demonstrated improved care quality and efficiency by reducing redundant tests and enabling timely, informed treatment decisions.

### Implementation resource team

The multi-disciplinary PC-TCM research team included PhD-prepared faculty, doctoral students, and administrative support representing the professions of nursing, social work, physical therapy, implementation science, industrial systems engineering, and computer science engineering. The team was divided into two sections, one supporting the technical aspect of the intervention and the other supporting implementation with the partnering organizations, joined through the HIE intervention. This paper focuses on the work of the implementation resource team (4) which was led by a nurse anthropologist, the principal investigator (PI), co-investigators with expertise in implementation science, systems engineering and social work, a project coordinator, and PhD students in nursing. This team worked intensively with the partners, both technical and clinical, through cross sector workshops, regular monthly meetings, *ad hoc* meetings over Zoom, and direct observation in the clinical sites. In addition to ethnographic observation and extensive fieldnotes, the team collected qualitative data in the form of periodic reflections from partners (managers and staff) (6) and semi-structured interviews with persons experiencing complex social and medical conditions. Following a participatory design approach, we involved frontline staff and administrators from all participating community sites. This process included engaging community partners early, so they were able to work closely with the implementation resource team across all phases of the design and testing of technological platforms.

**Figure 1:**
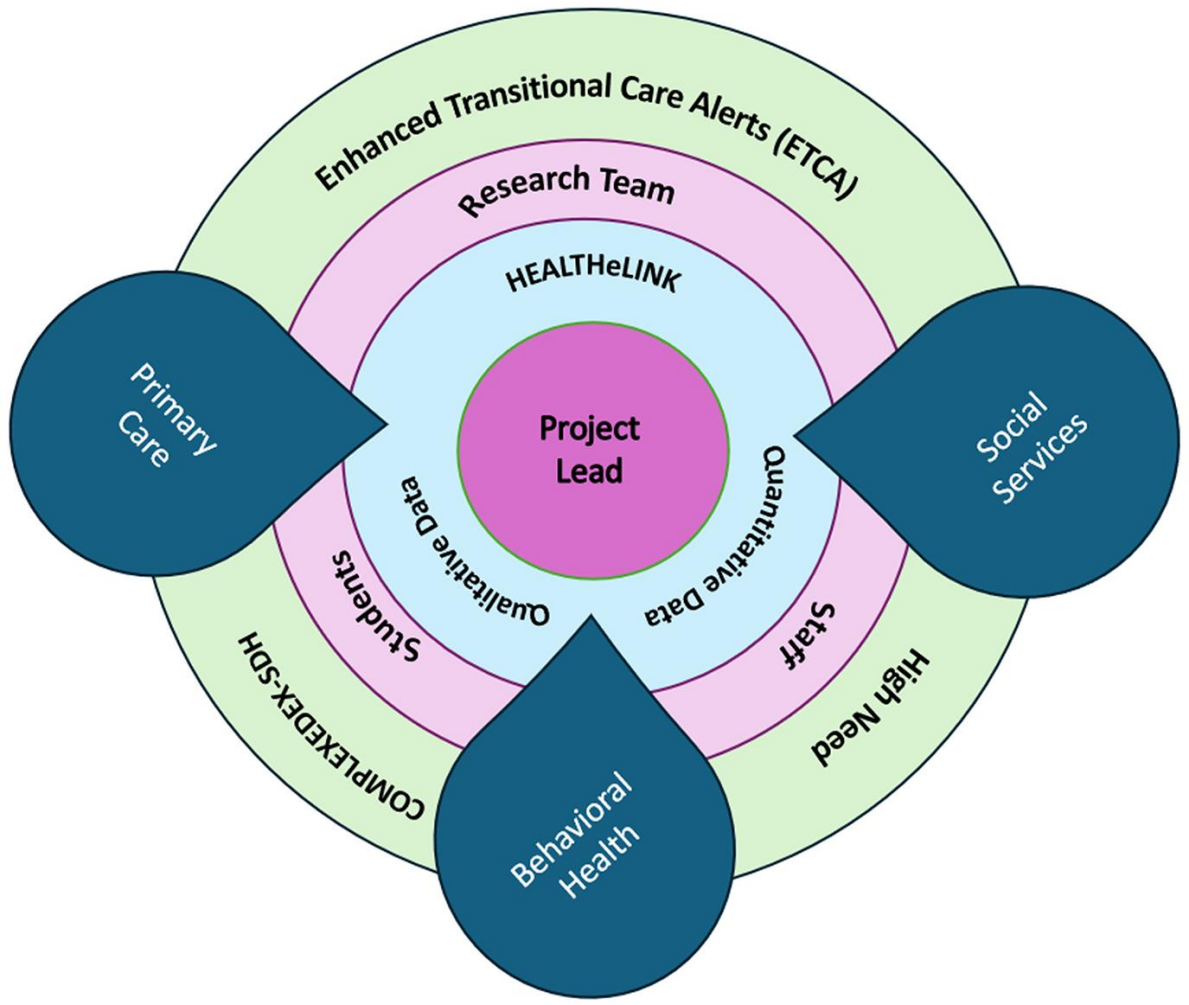
Overview of the relationships between the research team, community partners, data collection, and HEALTHeLINK.

To facilitate this process, meetings were held between clinical partners and the teams to closely monitor project milestone achievement and to share insights from ongoing data collection and analysis. The PI coordinated the project, led regular meetings, tracked milestones, and oversaw data sharing processes. Please see Figure 1 for a conceptual overview of the relationships between the research team, community partners, data collection processes, and the HIE.

### Tracking and reflecting on the implementation process

Throughout the project, we collected and reviewed data from 53 documents (2021–2024), including meeting minutes, transcripts of discussions, and project schedules to evaluate real-time decision-making processes, protocol deviations, near-misses (events potentially leading to implementation failure), and reflective insights on challenges and solutions encountered throughout the project. We also conducted qualitative interviews with research team members and clinical staff and technical staff at partnering organizations, alongside intensive interviews examining the perspectives of people with high medical, social and behavioral needs (6, 9) who were patients of the practice. The interviews were audio recorded; the data were transcribed, categorized, and qualitatively analyzed to identify patterns, themes, and relationships between the implementation process, barriers, and stakeholder roles. The findings of these qualitative interviews, including details regarding interviewee profiles, have been published elsewhere (10, 11).

### The socio-technical innovation

The project's innovation included both technical and social components. For the technical innovation, we developed an enhanced transitional care alert (ETCA), delivered electronically to primary care and behavioral health providers post-hospital discharge to optimize cross-sector collaboration. The ETCA relied on principles from health informatics, team science, and implementation theory, and HEALTHeLINK was central to the process, automatically pulling aggregated data into the ETCA from the primary care practice (practice roster and high needs report), the discharging acute care facility (discharge notification), and the clinical data repository to support timely outreach from the clinical site. For all patients receiving an admission discharge transfer (ADT) notification who also appeared in the high needs report, HEALTHeLINK aggregated these disparate data sources into a single ETCA. The ETCA information was automatically transmitted to the community-based organizations to facilitate cross-sector collaboration and shared care planning.

**Figure 2:**
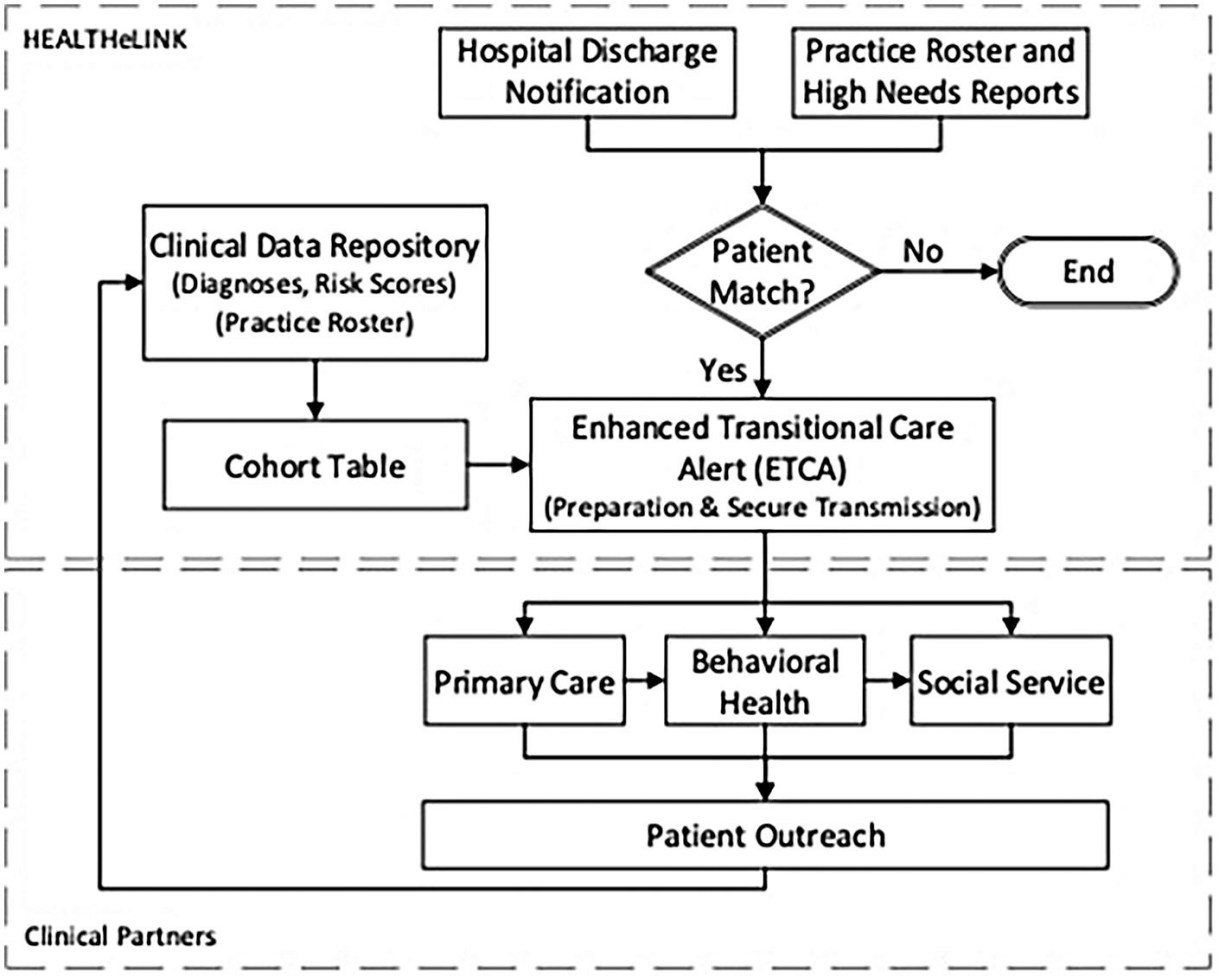
Information workflow. Note. Roster = Subscribe & Notify List (individuals who have consented to data sharing).

The social innovation involved an iterative process of stakeholder engagement and multi-disciplinary teams in the re-design and optimization of practice workflows in response to the ETCA. In this phase, the team worked closely with the clinical staff at the community sites to co-design a revised alert that included information that facilitated a nurse-led outreach phone call within 72 h of discharge. The final ETCA “dashboard” triggered by a hospital discharge, included: (1) contact and demographic information, (2) Adjusted Clinical Groups (ACG) Risk Score, (3) information from the admission, discharge, transfer (ADT) alerts, (4) information about all providers caring for the patient, and (5) relevant chronic/behavioral/social needs information (Figure 2).

### Targeting the high need population

We used overlapping qualitative, quantitative, and ethnographic approaches to better understand how to identify and classify individuals with high needs into useful ETCAs for the practice partners. First, we identified relevant codes and cross-walked them to the International Classification of Disease (ICD-10) for chronic conditions following the H-CUP Clinical Classifications Software Refined (CCSR) (12). Then, we developed a clinical algorithm for integrating social determinants of health (SDH) needs into the COMPLEXedex algorithm. The COMPLEXedex is a model for classifying risk based on a hierarchical ranking of 14 chronic health conditions (13–16). The COMPLEXedex-SDH, developed by nurses researchers on our team, integrated social needs identified through qualitative case analysis and classification of social needs was informed by the Gravity Project (17, 18). The COMPLEXedex-SDH identifies individuals with high needs based on having two or more chronic conditions, social risk factors, and care utilization patterns, shifting the focus from costs of care to overall need.

### Building relationships for sustained practice change

Ethnographic work included formal (workshops and meetings) and informal interactions (observations) with the clinical staff at the practices to inform development of the ETCA. These processes facilitated trust between stakeholders and the research team. By embedding themselves in a sustained, dialectical manner (19), the team helped bridge the persistent gap between academia and real-world implementation (20–22). The project coordinator facilitated communication centrally using email and cross-sector Zoom meetings to enable organizations to safely work out their idea collaboratively, while observing each other in the implementation process. This communication system was essential as both individuals and organizations were observed to be simultaneously espousing negative (this will never work), neutral (just doing the work), and positive (this is going to work great) sentiments, making parallel linear progress difficult. Initially, cooperation occurred without consensus (20), as participants struggled to align perspectives. However, repeated exposure to each other's positions allowed partners to develop shared understanding and collective goals, enabling the intervention to evolve to complexity in real time.

#### Failure to rescue

Originally a patient safety concept, “failure to rescue” refers to failing to recognize or respond to clinical deterioration (23–25). Factors influencing rescue success includes teamwork, taking-action, psychological safety, recognition of complications, and effective communication (22). In implementation, failure to rescue occurs when teams overlook or delay action on deviations from the planned implementation process, leading to incomplete or failed adoption. Preventing this requires both relational (trust, mutuality) and technical (expertise, reliability) strategies to support goals of the team (9, 26).

#### Implementation resource team

**Figure 3:**
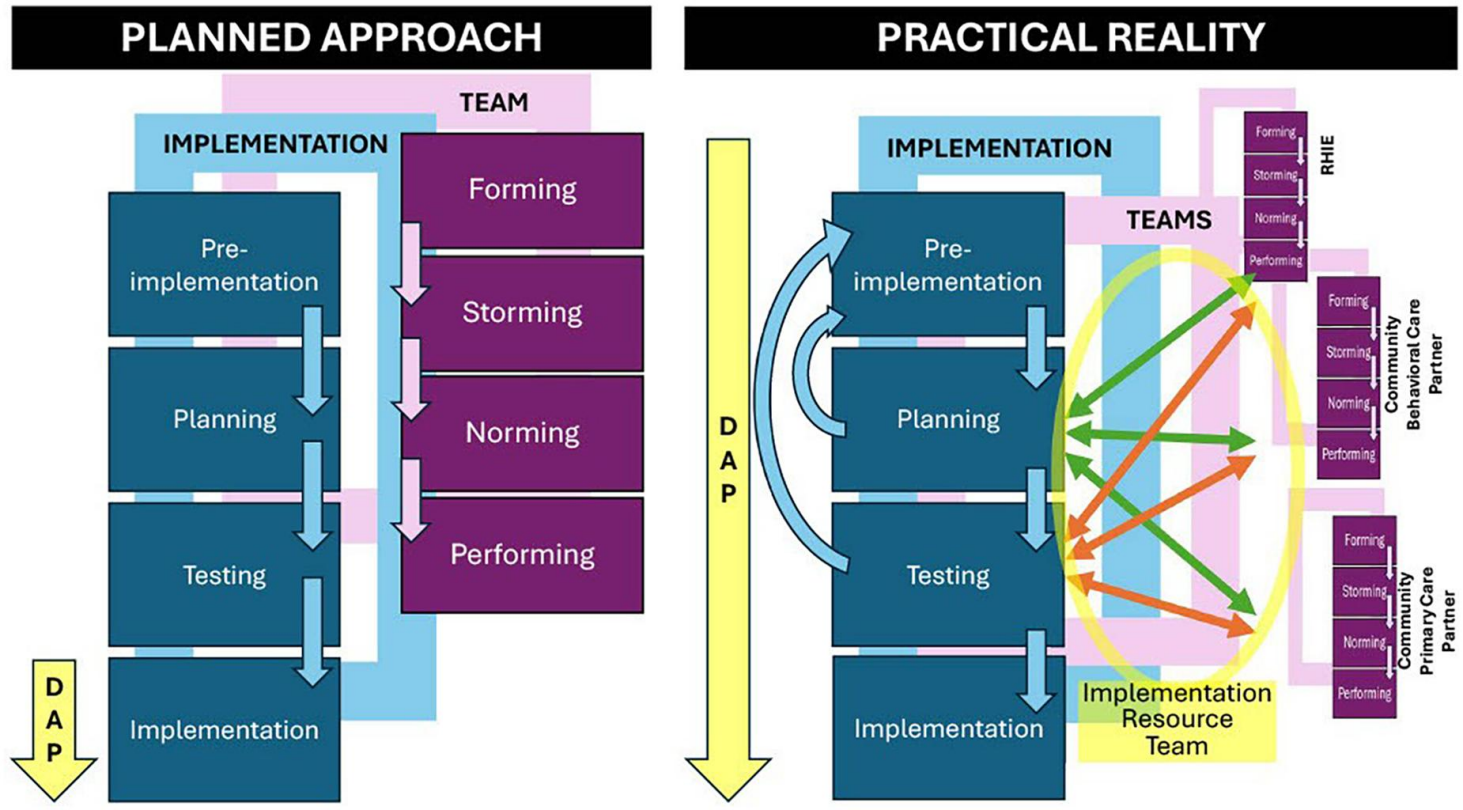
Extension of the dynamic adaptation process (DAP) model.

Similar to Rapid Response Teams in hospitals (27), an implementation resource team functions as a safeguard in complex, cross-sector settings to prevent implementation failure. The team includes implementation experts, multiple stakeholders and end users enabling real-time data monitoring, bi-directional communication, and adaptive problem-solving in real time, similar to the failure to rescue approach. The implementation resource team can play the role of the Rapid Response Team in complex, cross-sector implementation settings continuously monitoring the process, including identifying needs for adaptation, developing appropriate adaptation strategies, informing all stakeholders about changes, and guiding the processes. The resource team's continuous monitoring helped detect needed adaptations and minimized *ad hoc* changes that could threaten fidelity or lead to implementation drift (Figure 3).

## Results

This study contributes valuable knowledge about flexible implementation strategies that work well for supporting implementation of cross-sector socio-technical innovations for patients with complex medical, social, and behavioral needs. In this project, we identified several key implications for the implementation of complex, cross-sector socio-technical interventions in community settings: *Designing for Sustainability* and spread by engaging stakeholders from the initial design phase, *Engagement and Communication*, facilitated by a centralized communication system, *Understanding Baseline Organizational Readiness* during the pre-implementation phase, and *Holistic Design of Socio-Technical Interventions* through engagement of both community health partners and informatics engineers throughout the process (Table 1).

**Table 1:** Implications and lessons learned for the implementation of complex, cross-sector socio-technical interventions in community settings.

Theme	Key takeaways	Implications
Designing for Dissemination and Sustainability (D4DS)	The intervention's more rapid deployment at one of the community agencies, was largely driven by engagement and adaptations in the initial design phase, suggests that building a culture to address partners’ concerns from the outset can lead to smoother implementation and faster adoption.	Designing for scalability and spread should be a core consideration during the planning phase, not as an afterthought.
Engagement and Communication	The centralized communication system and regular meetings were crucial in allowing participating organizations to cooperate without immediate consensus. This flexible, non-linear approach helped organizations and individuals oscillate between negative, neutral, and positive perspectives, enabling them to better understand shared goals and perspectives.	Keeping collaborators involved, even during phases of low direct engagement, was essential for building trust and preventing failure to rescue.
Understanding Baseline Organizational Readiness	Pre-implementation efforts, such as interviews, and focus groups, were vital for understanding the baseline organizational culture, leadership dynamics, and stakeholder motivations.	These insights helped the research team anticipate potential barriers and guide intervention design to align with the needs and constraints of partner organizations.
Holistic Design of Socio-Technical Interventions	The importance of involving both community health partners and informatics engineers in all phases of the design, testing and implementation of technological platforms was critical for creating a sustainable care alert system and ensuring its acceptability and correct use by community partners.	A participatory design approach, which includes both frontline staff and administrators from all participating community sites, was salient for creating tools that fit with the organizational workflows and aligned with priorities of the stakeholders.

We present two case studies that illustrate how the implementation resource team applied the modified DAP to prevent failure to rescue.

### Case #1. Using patient perspectives to adapt the socio-technical intervention

Grounded in equitable implementation and designing for dissemination (5, 28), we prioritized integrating the patient experience [contending with the work of being a patient (“treatment burden”), self-care and self-management, medication management, and interacting with the healthcare system after hospital discharge] (29) into both the intervention and its evaluation. During planning, we interviewed 27 individuals from the practice sites with complex, co-occurring health conditions (e.g., diabetes, behavioral disorders) and unstable housing (10). Participants were recruited and interviewed at a social services organization offering shelter and medical respite care. Later, we interviewed 22 patients who received the nurse outreach calls in the immediate post-discharge period (11).

These conversations revealed that unmet social needs, particularly housing and food, often took priority over medical issues, and these unmet needs heavily influenced treatment burden (34). This information shaped all aspects of the intervention, from the ETCA content to nurse training content to ensure that outreach phone calls addressed social as well as clinical needs. Our findings also informed updates to standard treatment burden measures, ensuring relevance for patients with complex medical needs and social risk factors (11). These perspectives provided critical insights, enriching prior conceptual models, complementing data gathered from other stakeholders, and they shaped the development of the intervention and the implementation evaluation strategies. In complex cross-sector implementation environments, patient perspectives are a key information stream that can inform approaches to preventing failure to rescue the intervention, designing for sustainability, and avoiding implementation drift.

### Dynamic adaptation case #2: development of data-driven and actionable ETCA

Before the intervention, a nurse transitional care coordinator used ADT alerts during discharge follow-up, but they did not use the high-needs report in their calls. After gaining access to a list of the practice roster with flags for chronic conditions, we performed K-means clustering on a subset of patients with diabetes, identifying four subgroups (1) persons with comorbid substance use disorder, (2) chronic kidney disease or (3) language barriers, and (4) persons without those comorbidities with high medical and/or social needs (31). K-means clustering is a method that groups individuals into clusters based on how similar they are to each other. In healthcare settings, the K-means algorithm can help identify patient subgroups with similar needs so interventions can be better tailored to meet those needs (32). While we chose not to offer standardized care plans based on these groupings, we incorporated these insights into ETCA design to help nurses personalize outreach and care planning. Annual guided reflections with practice partners further enabled the resource team to closely monitor the implementation process, even when they were not actively participating in the intervention. This iterative, asynchronous process allowed ongoing experience to shape further adaptations and continued engagement of all stakeholders, consistent with the DAP framework.

## Discussion

This project proposes a structured approach for maximizing implementation success in the environment where ongoing changes take place. By borrowing from the literature on quality improvement, clinical safety, and software development agility, we adapted the DAP process to cover all phases of development, implementation and planning for sustainability of a socio-technical intervention. This approach allows for continuous adaptation of the intervention and implementation process and reduces the chance of implementation failure when designing complex socio-technical solutions in fast-paced healthcare environments.

To illustrate the new adaptation approach, we describe implementation of a socio-technical intervention designed to improve cross-sector collaborative care for patients with medical, social, and behavioral complexities (PC-TCM). The two case studies offer detailed insights into how unexpected changes in the implementation setting (“failure to rescue”) can be navigated to preserve implementation fidelity (“rescue the intervention”) and maximize intervention's fit-to-context. Key findings of the PC-TCM project include the central role of a communication system for disparate organizations to interact, overcome skepticism, and align goals, even when personnel changed.

The non-linear nature of collaboration, marked by fluctuations between positive, neutral, and negative attitudes towards team goals, initially hindered sequential implementation, but ultimately facilitated deeper understanding and cooperation between community practice partners. The study highlights the importance of *designing interventions for sustainability* and spread from the beginning, ensuring that collaborators remain engaged even when they were not directly contributing to the intervention. By facilitating care alert delivery via the HIE (HEALTHeLINK) with an algorithm that converted raw data into information and knowledge, the practice sites were also provided with actionable information and incentives to collaborate and improve patient care.

Previous research on improving implementation outcomes has focused on *a priori* identification and addressing of implementation barriers (33). However, it is impractical to expect these efforts to anticipate and prevent all situations of implementation failure, especially in the fast-paced environment of clinical care. Parallel attention must be placed on early signs and risk factors for implementation failure *during the intervention design and implementation process*. Our experience aligns with existing literature that acknowledges the non-linear and iterative nature of implementing complex interventions, especially when planning the intervention design phase (5). Despite efforts to accelerate implementation of clinical innovations, translation of research into practice requires continuous long-term implementation efforts (33).

Previous studies have also emphasized the importance of cross-sector communication and collaboration (20, 23, 30, 34, 35) for successful implementation of complex interventions similar to the PC-TCM project. This study provides new insights into the role of a centralized communication system during an implementation process that allowed for the simultaneous expression of diverse perspectives (negative, neutral, positive) and the eventual identification of shared values and goals guiding the implementation process. This process challenges prior understandings that suggest that collaboration requires immediate consensus (36). Thus, our findings emphasize the need for a sustained, cyclical approach to engagement to overcome barriers to implementation by emphasizing frequent stakeholder involvement and regular feedback on design, workflow impacts, and emerging needs.

The non-linear, adaptive implementation process helped bridge academic theory, the real-world environment, and implementation practices, allowing the organizations to navigate and adapt to the complexities of pragmatic collaboration in real time. Our findings suggest that building flexible infrastructures, fostering ongoing *engagement and communication*, and understanding the *baseline organizational readiness* and the landscape of partner organizations are critical for preventing failure to rescue with complex interventions. This study highlights the need for further research to develop validated methods for assessing implementation adaptation and to better understand the role of leadership and cross-sector collaboration in design and implementation of socio-technical innovations. Collaborative partnerships with national organizations and continued focus on the socio-technical dimensions of care delivery will be essential for refining implementation strategies and improving patient outcomes nationwide.

## Data Availability

The raw data supporting the conclusions of this article will be made available by the authors, without undue reservation.
